# Application of Nuclear Techniques to Improve the Mass Production and Management of Fruit Fly Parasitoids

**DOI:** 10.3390/insects3041105

**Published:** 2012-10-25

**Authors:** Jorge Cancino, Lía Ruíz, Mariana Viscarret, John Sivinski, Jorge Hendrichs

**Affiliations:** 1 Programa Moscafrut SAGARPA-IICA, Camino a los Cacahoatales S/N, 30860, Metapa de Domínguez, Chiapas, Mexico; E-Mail: lia.ruiz@iica-moscafrut.org.mx; 2 Insectario de Investigaciones para Lucha Biológica, Instituto de Microbiología y Zoología CICVyA, INTA, Castelar, 1712 Buenos Aires, Argentina; E-Mail: mviscarret@cnia.inta.gov.ar; 3 Center for Medical, Agricultural and Veterinary Entomology, Gainesville, FL 32608, USA; E-Mail: John.Sivinski@ars.usda.gov; 4 Joint FAO/IAEA Division of Nuclear Techniques in Food and Agriculture, A-1400 Vienna, Austria; E-Mail: J.Hendrichs@iaea.org

**Keywords:** irradiation, mass rearing, parasitoids, fruit flies, *Diachasmimorpha longicaudata*, *Anastrepha*, *Bactrocera*, *Ceratitis*

## Abstract

The use of irradiated hosts in mass rearing tephritid parasitoids represents an important technical advance in fruit fly augmentative biological control. Irradiation assures that fly emergence is avoided in non-parasitized hosts, while at the same time it has no appreciable effect on parasitoid quality, *i.e.*, fecundity, longevity and flight capability. Parasitoids of fruit fly eggs, larvae and pupae have all been shown to successfully develop in irradiated hosts, allowing a broad range of species to be shipped and released without post-rearing delays waiting for fly emergence and costly procedures to separate flies and wasps. This facilitates the early, more effective and less damaging shipment of natural enemies within hosts and across quarantined borders. In addition, the survival and dispersal of released parasitoids can be monitored by placing irradiated sentinel-hosts in the field. The optimal radiation dosages for host-sterility and parasitoid-fitness differ among species, and considerable progress has been made in integrating radiation into a variety of rearing procedures.

## 1. Introduction

Augmentative parasitoid/predator releases are an environmentally-friendly means of pest population suppression that are particularly useful when the pest has a greater rate of increase than its natural enemies and/or its populations begin to increase at times and places where natural enemies are not initially abundant [1,2]. Tephritid fruit flies are often such pests and large-scale releases of their parasitoids can contribute to suppressing their populations [3]. When integrated with the Sterile Insect Technique (SIT), natural enemies can support the sustainable development of “low-prevalence” and “fly-free” agricultural zones. 

While parasitoids, particularly opiine braconids, can sometimes inflict substantial mortality on frugivorous tephritids, classical biological control has often been insufficient [4]. In many cases this is because: (1) there are “refugia” for hosts, such as larger fruits in which fly larvae are beyond the reach of many parasitoids’ ovipositors [5]; (2) fruit flies tend to bridge gaps in host availability better than their parasitoids, and as a result escape early season suppression by their natural enemies [6]; and (3) naturally occurring parasitoid population densities cannot suppress pest populations to the minuscule levels required for commercial fruit and vegetable production and export [3]. These issues can be addressed to one degree or another through augmentative releases. For example, “refugia” can be breached by releases of larval parasitoids into patches of smaller fruit whose shallow pulp cannot shelter hosts or by the use of species that attack shallowly-buried eggs that are vulnerable in even the largest fruits. Mass-releases early in a fruiting season when natural enemies would otherwise be rare can prevent tephritid populations from growing and the combination of augmentation, sanitation, insecticide-bait sprays and the complementary SIT can result in commercially acceptable infestation levels [3]. The efficacy of augmentative releases in suppressing fruit flies (Diptera: Tephritidae) has been demonstrated in populations of *Ceratitis capitata* (Wiedemann) [7,8], *Bactrocera* spp. (Hendel) [9,10,11] and *Anastrepha* spp. (Schiner) [12,13].

Regardless of efficacy, what ultimately makes natural enemy augmentation economically viable is a cost effective means of mass rearing [14]. In this regard, fruit fly parasitoids have presented a number of challenges, some of which are now being overcome through the use of nuclear technology. Approximately a dozen programs successfully mass rear fruit fly parasitoid species [15,16,17], and technical advances in rearing have resulted in the routine production of millions of parasitoids/week. One of the techniques that have facilitated the development of fruit fly natural enemy mass rearing is the use of radiation to suppress the emergence of non-parasitized hosts. Pure parasitoid cohorts are yielded that can be released into the field without the risk of simultaneously releasing fertile flies [18,19]. This has proved to be particularly important when the emergence of adult hosts and parasitoids would normally at least partially overlap [20,21]. At the production level this facilitates host management, reduces rearing-steps, expedites transport and occasionally increases product quality [22,23,24,25,26]. At field sites storage and packaging for release is simplified and irradiated sentinel-host eggs and larvae can be used to monitor parasitoid survival and dispersal [14,25,27,28,29].

## 2. Background

Parasitism under mass-rearing conditions is never total and the separation of parasitoids prior to release can be both costly and often results in mechanical damage to the natural enemies [30]. In addition, parasitoid transport to field sites and other facilities is more effective and much easier within hosts than as adults. Historically, various methods had been proposed to separate parasitoids from hosts. For example, in some instances the developmental rates of wasps and flies were sufficiently different to allow adult hosts to emerge first, die and leave parasitoids to emerge alone from parasitized pupae [31]. However, in the majority of cases, some handling was necessary. Chemical growth regulators applied in host larvae to prevent fly adult development and to allow successful parasitoid emergence proved to be less than totally effective [32]. Host irradiation, which prevents successful completion of their development, is now routinely employed in the mass-rearing of several natural enemies [23,25]. The first application of irradiation for the production of a parasitoid was to preserve calyptrate fly pupae for subsequent exposure to parasitoids [33]. Irradiated pupae could be kept “on the shelf” for extended periods of time until needed as hosts for parasitoids of biting and “filth” flies that act as nuisances in dairy/livestock production. Since idiobiont ectoparasitoids of Diptera typically have broad host ranges, easily reared flies such as *Musca domestica* (L.) could be used to generate natural enemies and suppress target pests more difficult to culture [28,34].

An early attempt to use radiation in tephritid parasitoid mass rearing aimed to obtain sterile flies and parasitoids simultaneously. *Ceratitis capitata* pupae parasitized by *Diachasmimorpha* *longicaudata* (Ashmead) (Hymenoptera: Braconidae) were irradiated prior to adult emergence. Unfortunately, this treatment sterilized the parasitoids as well as the adult flies [35]. The first practical technique was developed by Sivinski and Smittle [18], who irradiated mature *Anastrepha suspensa* (Loew) larvae prior to parasitization by *D. longicaudata* and subsequently obtained homogeneous cohorts of fertile parasitoids. This successful experiment led to the widespread assessment and deployment of fruit fly parasitoids produced from irradiated hosts. 

However, there were problems of scale when irradiation was integrated into multiple mass rearing procedures. Not the least of these was presented by variable host volumes/densities within the irradiator. For example, while a thin layer of *Anastrepha ludens* (Loew) larvae exposed to 20 Gy can be used as *D. longicaudata* hosts, the dose needs to be increased to 40 Gy when a large volume of larvae (over a million) is irradiated [36]. Previous research into post-harvest phytosanitary treatments using radiation helped with calculations to determine the different doses needed to suppress egg, larval and pupal development of various Tephritidae [37]. Within this general framework it is possible to apply radiation not only to larval tephritids, but also to other host stages such as eggs and pupae, with adequate results regarding parasitoid emergence and effectiveness [19,38,39,40,41].

In all the aforementioned cases gamma radiation was applied. However, low-energy X-rays have recently also been used [42,43]. This type of radiation is emitted only when the electrical power is turned on and does not depend on radioactive isotopes with their associated risks and regulatory obstacles to their management and international transportation [44]. Mastrangelo *et al*. [42] compared the use of gamma and X-rays to induce sterility in *C. capitata* and *Anastrepha fraterculus* (Wiedemann), the South American fruit fly, and obtained 99% sterility in both species. Further studies on *C. capitata* and *A. fraterculus* exposure to X-rays [45,46] find that larval volume/density within the irradiation device affects even more than gamma radiation dosage formulations [19,44,47]. This kind of X-ray irradiation represents an important alternative technology in parasitoid mass rearing, but it will be necessary to reevaluate doses and methods.

## 3. Physiological Basis

Unstable elements (e.g., Cobalto-60, Cesium-137) produce ionizing radiation which decomposes into high energy ions emitted at low wavelengths. The emitted radiation is absorbed by any type of material which, as a result of the received energy, changes its chemical, physical or biological structure [44]. Ionizing radiation received by living organisms leads to oxidation chain reactions, forming peroxide free radicals that, depending on the dose, can cause irreversible alterations to molecules [48].

Radiation acts directly on the complex compounds of living organisms, the most affected are those in the process of formation or change [49]. As insects are subject to a series of major developmental/metabolic changes, they can be very susceptible to radiation [48], and its effect is greatest during metamorphosis. Thus radiation applied at an adequate dose and critical stage results in sterility, developmental suppression or other types of damage or physiological alterations [50].

In addition to the physiological state of the insect [51], body size is an important determinant of vulnerability to radiation with an inverse relationship between effective dosage and mass [52,53]. Thus in tephritid flies, the dose required to suppress development of the larger *A. ludens* larvae is actually lower than that required for the smaller *C. capitata* larvae [37,54].

In order for parasitoids to develop successfully on irradiated hosts, two important conditions must be met. First, the radiation cannot substantially diminish the quality of the host as a source of food [55]. Second, and this is particularly true for koinobiont endoparasitoids, the host’s interior physical and chemical conditions must still provide the cues and hormones required to orchestrate the parasitoid’s development [56]. Little is known about the nutritional and hormonal requirements of tephritid parasitoids. At this point in our research we can only deduce from the comparable quality of parasitoids raised on irradiated and unirradiated hosts that these requirements are not substantially violated by irradiation. There is even some tantalizing evidence that host-irradiation could enhance parasitism rates and parasitoid fitness [57].

Insects, including fruit flies, defend themselves against parasitoids through various immune mechanisms such as encapsulation [58,59,60,61]. In a majority of parasitoids, egg and first larval stage development is often very rapid [62], and voracious feeding early in their development may be a means acquiring critical resources before the host can mount a defensive response [63,64]. If radiation could compromise the host immune system, then a greater proportion of parasitoids might complete their development. It is known that radiation can damage the capacity of certain insect hosts to defend themselves and consequently a parasitoid may not confront fully competent resistance. For example, irradiation of the lepidopteran hosts of the braconid *Cotesia flavipes* (Cameron) (Hymenoptera: Braconidae) increased parasitism rates [24,65]. Some evidence likewise indicates that fruit fly larvae are immunologically compromised, thus radiation can result in a higher percent of parasitoid emergence. *Diachasmimorpha longicaudata* emergence and females-biased sex ratio increased following exposure of both *C. capitata* and *A. fraterculus* hosts to X-ray doses of between 20 Gy and 100 Gy [45,46]. Gamma irradiated *C. capitata* larvae also supported higher *D. longicaudata* emergence rates and produced a significantly greater proportion of females [66,67]. However, more studies are required to conclusively attribute increases in parasitism performance to reductions in host defenses.

Host age, for eggs and larvae, influences the effects of radiation. In both cases there is a combination of physiological and technical factors to consider when determining the optimal host age for irradiation and exposure to parasitoids. Timing of irradiation is particularly critical in the production of some tephritid pupal parasitoids. While irradiated host pupae are acceptable to them, when irradiated larvae are allowed to pupate they are usually no longer suitable hosts. Following irradiation the puparium cuticular layer forms but the pupa fails to develop properly, and pupal formation is critical, for different reasons, to both ecto and endoparasitoids. Ectoparasitoids of tephritid pupae oviposit into the space between the puparium and pupa [68], and in pupae derived from irradiated larvae this space does not develop [69]. In pupal endoparasitoids, *i.e.*, the diapriid *Coptera haywardi* (Oglobin), host unsuitability is related to biochemical changes resulting from radiation [70,71]. The puparium-pupal space also seems to be important to larval parasitoids of the family Figitidae, which fail to develop on irradiated hosts [72]. While these may be the only cases where emergence of adult parasitoids from irradiated fruit fly hosts have not been observed, their occurrence confirms that the physiological development of immature stages of parasitoids requires a combination of physical-chemical conditions that are not always present after host irradiation [73,74].

## 4. Optimizing Radiation Dose and Age of Irradiating Fruit Fly Hosts

The major variables in developing optimal mass-rearing procedures are: (1) radiation dose (Gy), (2) appropriate physiological age of the host (stage/instar/days) and (3) time of host exposure to radiation (Table 1). Success is measured by the number, quality and sex ratio of the resulting adult parasitoids as well as the complete suppression of fertile fly emergence. These variables have been identified in the mass rearing of a number of egg, larval and pupal hymenopteran parasitoids of tephritids.

**Figure 1:**
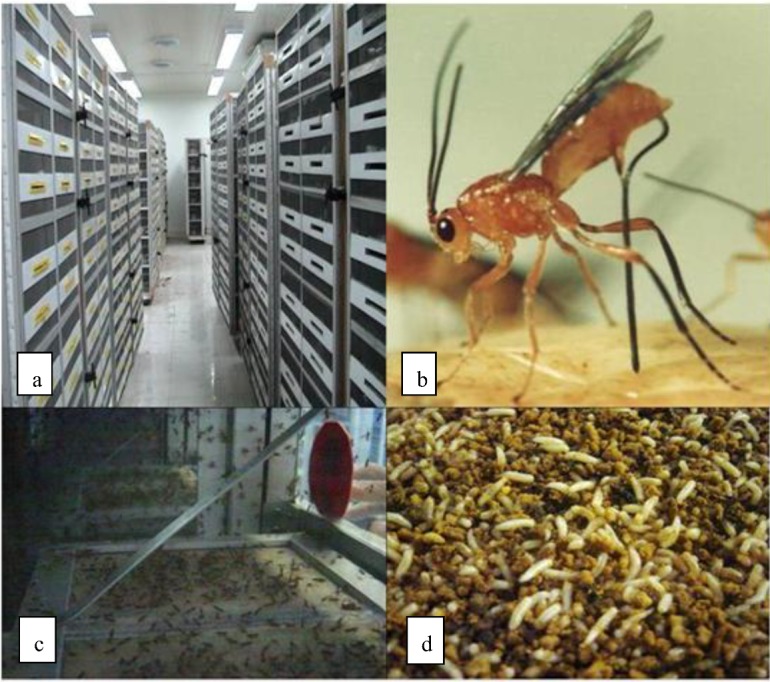
Mass rearing of *D. longicaudata* with irradiated host larvae. Moscafrut Program, México. (**a**) Cages in the adult colony, (**b**) *D. longicaudata* female ovipositing, (**c**) an adult parasitoid rearing cage, (**d**) larvae that have been exposed to parasitoids, including pupated larvae.

Development in irradiated hosts has been described for 10 species of Braconidae (Opiinae). One of these, *Fopius arisanus* (Sonan), originally from tropical Asia, is an important egg-prepupal parasitoid for the control of *Bactrocera* spp. and *C. capitata* [75,76]. The remaining species attack larvae and are native to Asia/Indo-Australian or the Neotropics [7,77,78]. *Diachasmimorpha longicaudata* deserves special mention, as it is widely used to control *Anastrepha* spp., as well as *Bactrocera* spp. and *C. capitata* (Figure 1) [78,79]. Species of the *Psyttaliaconcolor* (Szépligeti) complex are interesting [80] since they can be reared on the factitious host *C. capitata* to produce parasitoids for the control of the olive fly *Bactrocera oleae* (Gmelin) [81,82]. *Bactrocera tryoni* (Froggatt) is an unusual host in that irradiation arrests its development to the point that emergence of the parasitoid (*Diachasmimorphakraussii* (Fullaway) is compromised [83].

**Table 1:** Host stages, instars and radiation dosages used in the mass-rearing of various egg, larval and pupal hymenopteran parasitoids of tephritids under different host and irradiator conditions.

Family	Parasitoid species	Host species	Stage (instar)	Irradiation Dose (Gy)	Host irradiation	Irradiator / conditions	Reference
Braconidae	*Fopius arisanus*	*Anastrepha* *ludens*	Egg ^1^	27.5	1 mL egg	Gammacell 220 Co 60	[38]
					3 mL of water	2.3–3.0 Gy/min free oxygen	
	*Diachasmimorpha longicaudata*	*Anastrepha* *ludens*	Egg ^1^	27.5	1 mL egg	Gammacell 220 Co 60	[38]
					3 mL of water	2.5–3.0 Gy/min free oxygen	
		*Anastrepha* *ludens*	Larva (3rd)	20	100 larvae	Gammacell 220 Co 60	[19]
					naked	2.5–3.0 Gy/min free oxygen	
		*Anastrepha obliqua*	Larva (3rd)	30	100 larvae	Gammacell 220 Co 60	[19]
					naked	2.5–3.0 Gy/min free oxygen	
		*Anastrepha* *serpentina*	Larva (3rd)	20	100 larvae	Gammacell 220 Co 60	[19]
					naked	2.5–3.0 Gy/min free oxygen	
		*Anastrepha* *suspensa*	Larva (3rd)	20	200 larvae	137 Cs source	[18]
					naked	1732 roentgens/min	
		*Ceratitis* *capitata*	Larva (3rd)	60–65	larvae	Gammabean 650 Co 60 type IR31	[66,67]
					naked	226.9–287.83 Gy/h	
	*Doryctobracon* *crawfordi*	*Anastrepha* *ludens*	Larva (3rd)	20	100 larvae	Gammacell 220 Co 60	[39]
					naked	2.5–3.0 Gy/min free oxygen	
	*Doryctobracon* *aerolatus*	*Anastrepha suspensa*	Larva (2nd)	70	Larvae mixed	Gammacell 1,000 Cs137	[84]
					in diet	8.9 Gy/min	
	*Opius* *hirtus*	*Anastrepha* *ludens*	Larva (3rd)	20	100 larvae	Gammacell 220 Co 60	[39]
					naked	2.5–3.0 Gy/min free oxygen	
	*Utetes* *anastrephae*	*Anastrepha* *ludens*	Larva (3rd)	20	100 larvae	Gammacell 220 Co 60	[39]
					naked	2.5–3.0 Gy/min free oxygen	
	*Diachasmimorpha* *tryoni*	*Ceratitis capitata*	Larva (3rd)	40	100 larvae	Gammacell 220 Co 60	[19]
					naked	2.5–3.0 Gy/min free oxygen	
	*Psyttalia* *concolor*	*Ceratitis capitata*	Larva (3rd)	60	Larvae in water	Theratron Co 60	[41]
						type C-146; 107.33 cGy/min	
	*Psytalia* *humillis* **	*Ceratitis capitata* **	Larva (3rd)	70	1 Lt naked larvaein a plastic bag	Gammacell 220 Co 60 3.0 Gy/min	[81]
	*Diachasmimorpha* *kraussii*	*Bactrocera* *tryoni*	Larva (2nd–3rd)	15.9-26.8	Larvae with dietin Petri dishes.	Gamma Technology Research Irradiator Co 60	[83]
Eulophidae	*Aceratoneuromyia indica*	*Anastrepha ludens*	Larva (3rd)	45	100 larvae	JS-120 Co 60	[85]
					naked	4.22 Gy/min	
Diapriidae	*Coptera* *haywardi*	*Anastrepha ludens*	Pupa ^2^	20	100 pupae	Gammacell 220 Co 60	[39]
					naked	2.5–3.0 Gy/min free oxygen	
Eurytomidae	*Eurytoma* *sivinski*	*Anastrepha ludens*	Pupa ^2^	20	100 pupae	Gammacell 220 Co 60	[39]
					naked	2.5–3.0 Gy/min free oxygen	
Chalcidoidea	*Dirhinus* spp.	*Anastrepha ludens*	Pupa ^2^	20	100 pupae	Gammacell 220 Co 60	[39]
					naked	2.5–3.0 Gy/min free oxygen	

^1^ Eggs exposed to radiation at the age of 72 h. ^2^ Pupae exposed to radiation at the age of 3–5 days.

A koinobiont outside of the Braconidae, the eulophid *Aceratoneuromyia indica* (Silvestri), can develop in irradiated hosts and attacks larvae in various genera of Tephritidae (*Bactrocera* spp., *Anastrepha* spp.). Little is known about the developmental biology of this species [78], however, it could be important to biocontrol since it searches for host larvae inside infested fruit and may not find large fruit size a barrier to oviposition [86].

Pupal parasitoids have also been considered for tephritid biological control and can be divided into two groups that may differ in their developmental responses to host irradiation (see Section 3, Physiological Basis). The first group is composed of species of *Dirhinus* (Chalcididae) and *Eurytoma sivinskii* (Eurytomidae) (Gates and Grissell). These are solitary ectoparasitic idiobionts and generalists that develop in a wide range of Diptera [87,88]. The second consists of endoparasitoids of the family Diapriidae, particularly *C. haywardi.* These appear to be specialized on Tephritidae and so are likely to have less effect on non-target species and to remain focused on declining pest populations [89]. 

The emergence of adult parasitoids from their host puparia requires removal of host larvae from the potential contaminants in the artificial diet and their placement into a suitable pupation environment. From an operational perspective, host irradiation poses few adverse effects in this regard. An exception is that irradiation of *Bactrocera dorsalis* (Hendel) second instar larvae, used for mass rearing of *Fopius vandenboschi* (Fullaway), prevents mature larvae from jumping in order to exit diet trays. This creates a problem for parasitoid mass-rearing since there is no equally efficient artificial technique to remove larvae from diet (Kuswadi, personal communication).

The micro-environmental conditions under which radiation is applied are important to its effectiveness. For instance, Cancino *et al*. [19] irradiated *C. capitata* third instar larvae directly without any additional substrate (diet, vermiculite, water *etc*.), while Hepdurgun *et al*. [41] irradiated larvae of similar age covered in water. Since water is an effective radiation barrier [90], Hepdurgun *et al.*’s larvae required an additional 20 Gy to be sterilized.

To an even greater degree than gamma rays, the efficacy of X-rays depends upon the manner of host presentation. For example, a dose of 60 Gy applied to 150–200 third instar *C. capitata* placed in a Petri dish, completely inhibits adult fly emergence, whether or not larvae are immersed in diet. However, adult development and eclosion of ~19,000 larvae in a larger container was not completely suppressed even with a dose 100 Gy [45]. X-rays have lower penetration than gamma rays [43] and their efficiency is greatly reduced when larvae are immersed in diet and presented in deep-bodied trays. Equivalent doses of gamma and X-rays induced sterility in *A. fraterculus* [46] but this might have been due to small numbers of larvae (approx. 2,200) being presented in shallow diet trays (Table 2).

**Table 2:** X-ray doses applied in two fruit fly species for mass rearing the hymenopteran larval parasitoid *D. longicaudata*.

Host Species	Stage (instar)	Irradiation Dose	Host Irradiation	Irradiator/conditions	Reference
*Ceratitis capitata* **	Larva (3rd)	6,250.2 R (60 Gy)	150–200 larvae with and without larval diet	Philips MG 160 Constant Potential X-ray System-Minus H:T. Generator Type 160 kV/4 kW Free oxygen	[45]
*Anastrepha fraterculus*	Larva (3rd)	10,417 R (100 Gy)	2,200 larvae mixed in diet	Philips MG 160 Constant Potential X-ray System-Minus H:T. Generator Type 160 kV/4 kW Free oxygen	[46]

In facilities mass rearing parasitoids for augmentative releases, large quantities of hosts are treated in each radiation session, and optimal radiation doses for particular scales of irradiation and hosts have been experimentally derived [36]. Unfortunately, this process has only occasionally been put into practice and then only for some parasitoid species. Table 3 lists the reported doses used to irradiate large quantities of hosts. The doses applied are universally higher than those suggested in the literature as necessary to prevent fly emergence (see Table 1).

**Table 3:** Gamma ray doses used for irradiation of hosts in the large-scale mass rearing of fruit fly parasitoids.

Parasitoid species	Host species	Stage (instar)	Irradiation Dose (Gy)	Irradiator/conditions	Weekly Pupae Production	Reference
*D. longicaudata*	*A. ludens*	Larvae (3rd)	45	JS-120 Co 60	50 millions	[19,91]
				4.22 Gy /min		
*D. longicaudata*	*A. suspensa*	Larvae (3rd)	40	Gammacell 1,000 Cs 137	~150,000	[12]
				12 Gy/min		
*D. krausi*	*C. capitata*	Larvae (3rd)	70	Gammacell 220 Co 60	~1 million	[92]
				3 Gy/min		
*D. tryoni*	*C. capitata*	Larvae (3rd)	70	Gammacell 220 Co 60	~1 million	[93]
				3 Gy/min		
*P. humillis*	*C. capitata*	Larvae (3rd)	70	Gammacell 220 Co 60	~100,000	[81]
				3 Gy/min		
*C. haywardi*	*A. ludens*	Pupae	40	JS-120 Co 60	150,000	[17,39]
				4.22 Gy/min		

## 5. Quality of Emerged Adult Parasitoids

While the numbers of parasitoids reared and the complete removal of fertile flies are important to augmentative biological control, it is equally important that the parasitoids produced retain the foraging and reproductive abilities that make them effective biological control agents. Some important parameters to measure production quality, such as pupation, adult emergence, sex ratio, longevity, fecundity and flight capability, have been established [94,95]. One comparison of *D. longicaudata* reared on irradiated and non-irradiated hosts found no significant differences in the mentioned parameters except for a lower rate of pupation in those larvae that had failed to mature within 72 h of irradiation [19]. 

A mixture of developing flies and parasitoids in the same pupation medium can decrease parasitoid survival. Flies increase micro-environmental temperatures which can result in degraded hygienic conditions and poorer parasitoid health. Exclusive production of parasitoids reduces mortality and improves emergence, biases sex ratios towards females, and improves longevity and flight capability [96].

## 6. Practical Applications

**Figure 2:**
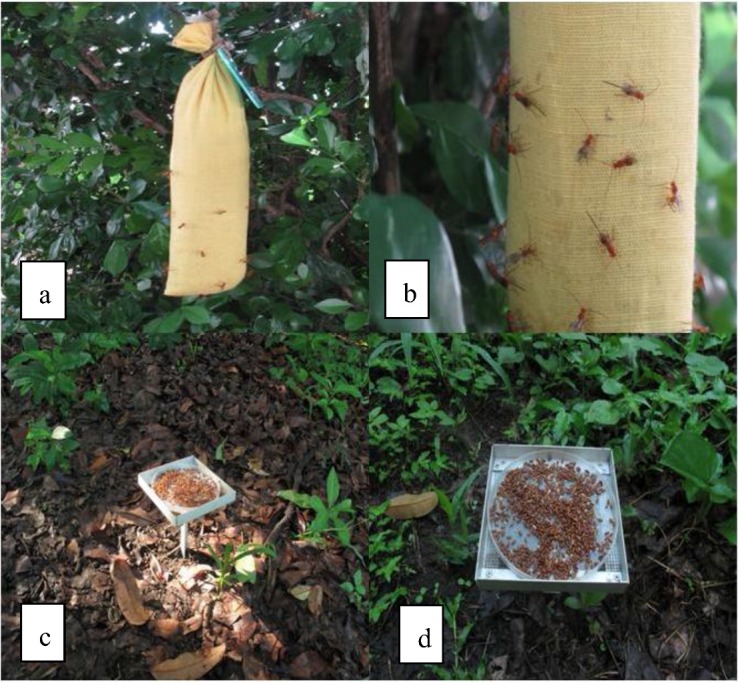
Different devices for evaluating parasitoid presence or activity in the field using irradiated host larvae or pupae. (**a**) “Sausage” trap with 200 irradiated larvae and diet for *D. longicaudata* evaluations, (**b**) *D. longicaudata* females ovipositing into hosts within the trap, c and d) two views of traps with approximately 1,000 irradiated pupae and vermiculite used for evaluation of pupal parasitoids.

The use of irradiated hosts has improved the rearing-efficacy of fruit fly parasitoids and perhaps their quality as biological control agents. The following are among the most important of radiation’s specific contributions:

(a) **Avoidance of host emergence:** This is without doubt the most important consequence of host irradiation. Developmental suppression of non-parasitized hosts, which represent between 10%–50% of hosts under mass rearing conditions, is a key to increasing parasitoid production to the level of millions per week. Without irradiation it is practically impossible to maintain large-scale production without the risk of shipping and releasing pest flies.
(b) **Increased production:** As a result of suppression of host defenses, irradiation of the hosts can in some cases increase parasitoid emergence rates [66,67]. Also, host mortality is reduced and parasitoid emergence increased as a result of larval or pupal medium sanitation.
(c) **Pupae packing and shipment:** Production laboratories are often located far from the targeted release areas. The transportation of exclusively parasitized pupae in plastic bags under hypoxic conditions improves security considerations and so facilitates transport, handling and makes post-transport quality evaluations simpler to perform [97].
(d) **Preparation for release:** The sole emergence of adult parasitoids facilitates the design of methods to release millions of parasitoids in the field [12,13]. This is particularly true when devices such as those employed for aerial release need to be calibrated for a particular size of insect with unique environmental tolerances [93,97].
(e) **Parasitoid quality:** Avoiding the separation of parasitoids and flies, allowing the packing and transport of only parasitoids in pupae rather than as adults, significantly increases their quality. Also, fewer dead host larvae and pupae during the production phase as a result of the exclusive development of parasitoids, improves sanitation and the quality of mass produced parasitoids while favoring a female-biased sex ratio [66,67,96].
(f) **Field evaluations:** Parasitoid presence, behavior, survival and dispersal can be assessed by deploying devices baited with irradiated hosts (Figure 2) [13]. Irradiated hosts do not present an infestation risk in the field. The use of such devices is currently the only practical alternative to experiments conducted under laboratory or greenhouse conditions [98].
(g) **Export of parasitoids within quarantined pest pupae:** The MOSCAFRUT production facility in Mexico has exported *D. longicaudata* parasitoids to different countries such as Argentina, Brazil, Colombia, Costa Rica and Peru [79,99]. This involved the transport of *A. ludens* as the host, a quarantined fly species in all the mentioned countries. The consignments were carried out using parasitoids in irradiated hosts, thus eliminating the risk of introducing an economically important species. In addition, the Campaña Nacional contra Moscas de la Fruta (National Campaign against Fruit Flies) in Mexico, transports millions of *A. ludens* pupae containing *D. longicaudata* weekly to various “low prevalence” agricultural production zones in northern Mexico. Since these are areas where eradication or suppression campaigns are ongoing, the inadvertent release of fertile flies would be disastrous [91]. However, due to host irradiation there have been no reports of fruit fly contamination in over 15 years. Another noteworthy case is the import and release of *Psyttalia humilis* (Silvestri) in California for the control of *B. oleae*. These parasitoids are produced using irradiated *C. capitata* [81] larvae transported from Guatemala. This species of fly is commercially important throughout the world and is a quarantine species in the United States. Without irradiation, the project in its present form would not be feasible.

## 7. Conclusions

Augmentative biological control has a promising future as part of integrated fruit fly management. Assessments carried out in the field have demonstrated the effectiveness and advantages of the approach. The availability of many parasitoid species provides a much wider scope of biological control alternatives than in the past to manage different fruit flies under different situations and climatic conditions. However, there is still need for more research to further reduce mass rearing costs, including the production of irradiated hosts. For example, X-ray radiation devices and techniques require further evaluation in order to determine the best doses and host physiological states.

The use of irradiated hosts in mass rearing is essential for large-scale management of parasitoids. However, a significant constraint is access to radiation sources. These are costly [44], thus their use is largely restricted to large commercial producers [100] or to government institutions that support area-wide pest management approaches. The advantages of irradiation will continue to drive a demand for alternative X-ray devices that more cost-effective and for studies of parasitoid physiology, behavior and other biological attributes in support of augmentative biological control. For example, the use of irradiated eggs for the development of larvae that can be used as hosts of *D. longicaudata* or other larval parasitoids could reduce handling time, because it is faster and easier to irradiate large numbers of eggs. Also, easily reared factitious host larvae and pupae could lower mass production costs.

The mass rearing of fruit fly parasitoids requires the infrastructure for the mass production of hosts, which means that initial investment costs are high. This has led some regional control programs to procure parasitoids from other production centers. As this requires the elimination of the risk of introducing quarantine pest species, the use of irradiated hosts for transboundary movement will increasingly become compulsory. 

In the field, evaluation of parasitoid efficiency can be further developed by means of devices with irradiated host eggs, larvae or pupae [24,29]. Studies of this type have already provided interesting information on the dispersal of released parasitoids. In addition to monitoring parasitoid presence and activity, irradiated hosts could be placed in the field early in the fruiting season to build up natural parasitoid populations. Such an approach would be foolhardy without radiation technologies.

In conclusion, the use of irradiated hosts is fundamental for the production of fruit fly parasitoids. It not only facilitates augmentative biological control, it also creates opportunities for other novel environmentally-friendly control techniques that can join augmentation in an integrated approach to area-wide fruit fly management.
